# High resolution Raman spectroscopy mapping of stem cell micropatterns†

**DOI:** 10.1039/c4an02346c

**Published:** 2015-03-21

**Authors:** Thomas C. von Erlach, Martin A. B. Hedegaarda, Molly M. Stevens

**Affiliations:** a Department of Materials, Department of Bioengineering and Institute of Biomedical Engineering, Imperial College London, London SW7 2AZ, UK; b Department of Chemical Engineering, Biotechnology and Environmental Technology, Niels Bohrs Allé 1, 5230 Odense M, Denmark

## Abstract

We report on the use of high resolution Raman spectroscopy mapping combined with a micro-engineered stem cell platform. This technique obtains quantitative information about the concentration of individual intracellular molecules such as proteins, lipids, and other metabolites, while tightly controlling cell shape and adhesion. This new quantitative analysis will prove highly relevant for *in vitro* drug screening applications and regenerative medicine.

## Introduction

The focus of stem cell research has traditionally been on soluble factors such as cytokines and growth factors to direct stem cells into one specific cell type for the regeneration of diseased or damaged tissues. However, the importance of physical cues in modulating stem cell function has recently become more apparent as a critical regulator of cell fate.^1^–^7^ Research in this field has been driven by the maturation of micro-engineering techniques that have enabled the confinement of cells into defined shapes *in vitro*. Such techniques have allowed for an examination of the effects that changes in architecture have at the single cell level. These new techniques have brought to light the fundamental importance of the cell’s cytoskeleton in sensing changes in the microenvironment. This information is then translated inside the cell, allowing it to rapidly adapt and respond to the changing microenvironment. Several studies produced of cellular micropatterns have investigated numerous aspects of cell physiology including motility, division, polarity, cytoskeleton dynamics, and stemness.^8^–^14^ Therefore, it is of high interest to decipher the molecular processes that translate intrinsic information generated by cell architecture to downstream signalling effects. Additionally, it is of fundamental interest to investigate the changes associated with a cell’s molecular composition and organisation following alterations in architecture. This new information could provide previously unexplored insights into signalling mechanisms that are relevant for the development of novel drug targets and cell therapies. Current techniques to analyse cell micropatterns are almost all exclusively based on immunohistochemistry.^3^,^4^,^14^ Taking advantage of the large number of individual cells in identical conditions within the cell micropatterns, it is important to develop analytical techniques that shed light on the intracellular concentration and localisation of a broader range of molecules. Extending the analysis to molecules beyond those labelled by immunohistochemistry before analysis increases the amount of information generated and holds the potential to examine the impact of change and effect. Raman micro spectroscopy mapping is a label free technique based only on molecular vibrations that can provide quantitative insights into protein and lipid distribution, and in some cases the distribution of specific molecules.^15^–^18^ Raman micro spectroscopy provides label-free information from a small volume within the cells in high-resolution, with greater than 500 nm resolution following visible laser excitation. Using multivariate analysis methods, it is possible to extract the spatial distribution of molecular components such as proteins, lipids, and nucleic acids. In addition, Raman micro spectroscopy can perform label-free live cell imaging and could potentially analyse the dynamics of molecules and structure in a micropatterned cell over time.^19^–^21^

## Results and discussion

### Development of cell micropattern arrays

**Fig. 1:**
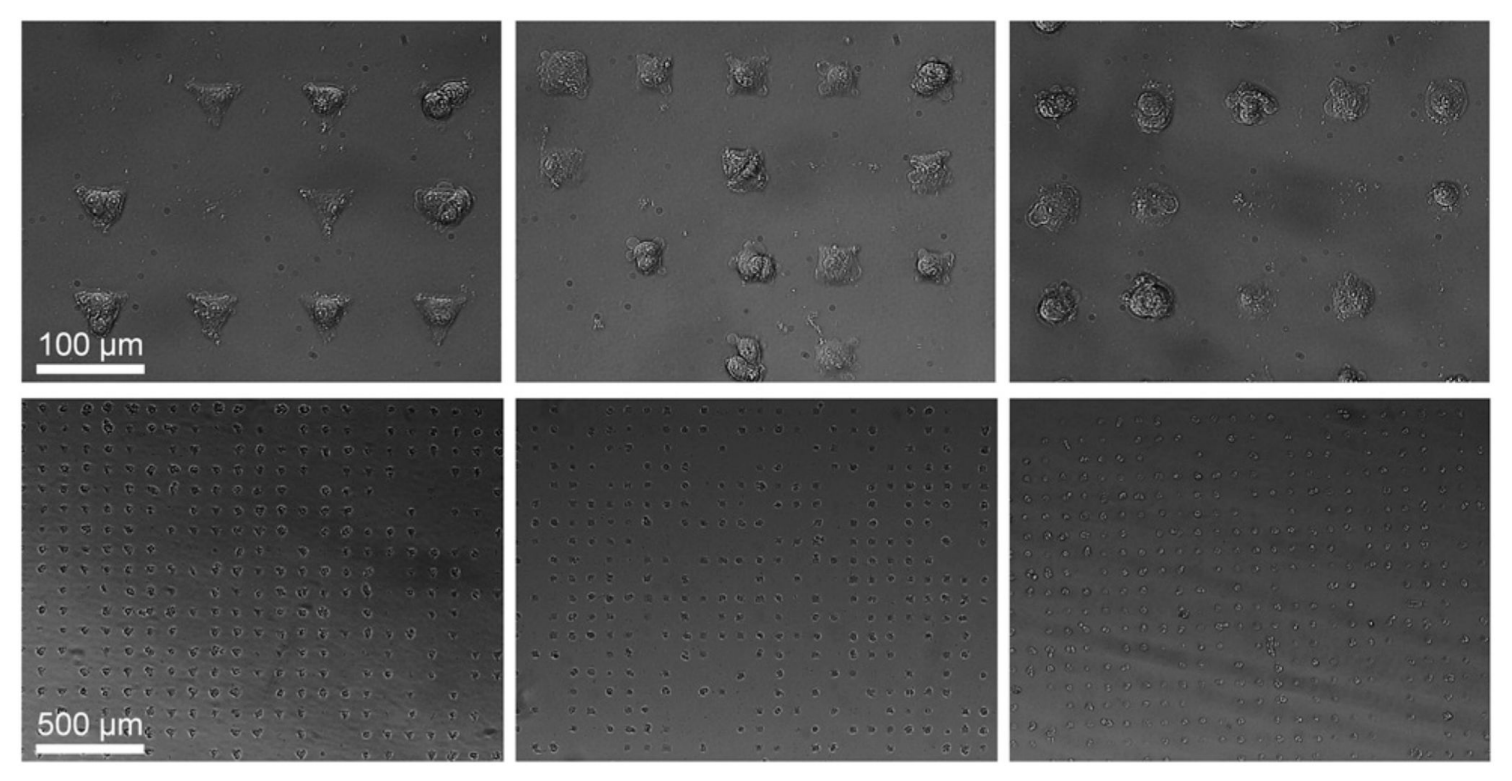
Micropatterned hMSCs after 24 hours incubation. Cells adapt to the underlining shape of the FN micro-islands resulting into triangular, square, and circular shaped cells. The islands have an identical cell adhesion area of 1350 µm^2^ but a different cellular architecture.

Micropatterned glass substrates containing fibronectin (FN)-coated islands were fabricated using a modified literature protocol for micro-contract printing.^22^ Stamps were made by replica casting polydimethylsiloxane (PDMS, Sylgard 184; Dow Corning, Midland, MI) against a silicon master made by photolithography (gift from Marcus Textor). PDMS pre-polymer (1 : 10, curing agent to prepolymer) was poured over the silicon and cured at 60 °C overnight. The elastomeric PDMS stamp bearing the negative pattern of the master was peeled off and stored dry in a closed well plate at room temperature. Stamps were sonicated for 30 minutes in ethanol, rinsed three times with distilled water, dried under nitrogen, oxidised in air plasma for 1 minute (200 mtorr) (Plasma Prep 5, Gala Instruments), and used for contact printing immediately. To allow the adsorption of proteins, plasma-activated stamps were immersed for 1 hour in an aqueous solution of bovine FN (50 mg mL^−1^, Sigma). Stamps were dried under compressed air and placed in conformal contact with the substrate (non-treated polystyrene multi-dish, Nunclon Surface) for 60 seconds before being peeled off. Subsequently, substrates were immersed in 0.1% (w/v) Pluronic F127 (Sigma) in phosphate buffered saline (PBS) for 1 hour and carefully rinsed with water without allowing the surface to dry. The produced micropatterns were either used immediately or stored up to two days at 4 °C in PBS. For cell culture, micropatterns were pre-incubated in 10% (w/v) antibiotics in PBS. After three times washing with PBS, 15 000 cells per cm^2^ were seeded in serum free medium. Human mesenchymal stem cells (hMSCs) (PromoCell) were cultured in hMSC growth medium (PromoCell) under standard cell culture conditions (37 °C, 5% CO_2_). Cells were detached using 0.25% (w/v) Trypsin-EDTA solution (Invitrogen) and seeded in serum free growth medium at a density of 13 000 cells per cm^2^ on the micropatterned substrate. hMSCs seeded on top of these substrates attached to these regions and spread to assume the shape of the underlying island. We constructed triangular, square, and circular shaped FN micro-islands with an identical surface area of 1350 µm^2^ (Fig. 1).

### Raman spectroscopy analysis

**Fig. 2:**
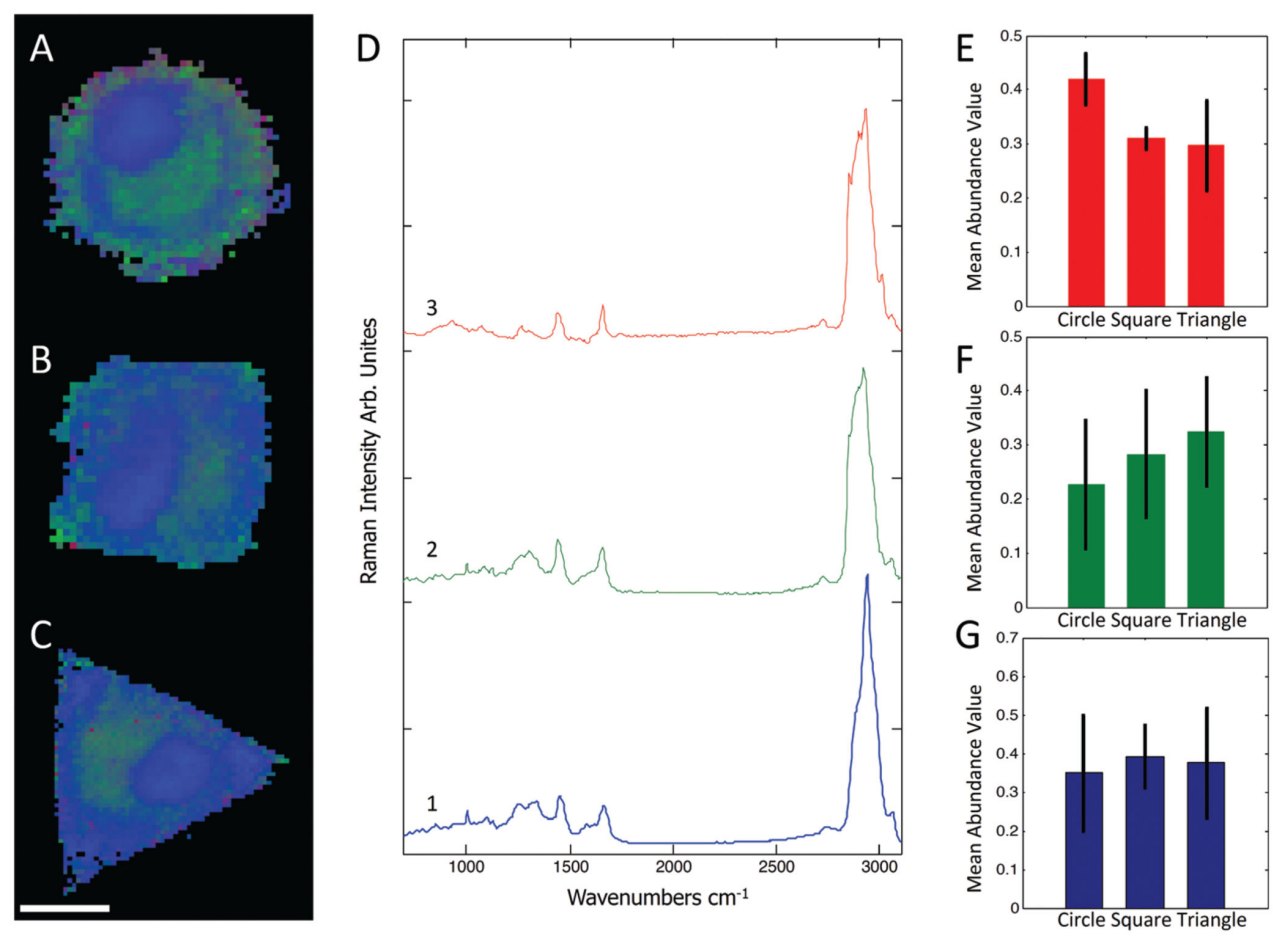
Representative combined N-FINDR images of circular (A), square (B), and triangular (C) cells. The red spectrum (D,3) indicates lipid content, green (D,2) is assigned as cytoplasm, and blue (D,1) as nucleus spectra. The mean abundance value for each selected endmember is shown in (E–F), which suggested a higher lipid content in circular cells as opposed to a higher protein content in square and triangular cells. Scale bar = 20 µm.

Raman spectra were collected using a Renishaw RM2000 (Renishaw, Wotton-under-Edge, Gloucestershire, UK) dispersive spectrometer system connected to a Leica microscope equipped with a Renishaw MS20 motorised stage. A 514.5 nm Argon Ion laser (Melles Griot, Albuquerque, NM) with 130 mW at source was used for excitation, producing ~60 mW at sample. Raman spectroscopy images were collected using 1 second integration time per spectrum and a step size of 800 nm using a Nikon 60×/NA = 1.0 (Nikon, Kingston upon Thames, UK) water immersion objective. A total of 8–12 micropatterned single hMSCs from independent cultures for each micro-island shape were measured. hMSCs were fixed with 4% (v/v) formalin in dH_2_O (Sigma) for 15 minutes at room temperature, washed PBS three times, and stored at 4 °C for maximum 3 days before being analysed. Images were analysed using in-house written MatLab (The Mathworks, Natick, MA). Each image was corrected for water and glass backgrounds using Extended Multiplicative Scatter Correction with Spectral Interference Subtraction (EMSC-SIS), and the low signal-to-noise spectra surrounding the cell were removed.^23^ The method normalised the dataset using EMSC and then subtracted the backgrounds of water and glass according to a best spectrum extracted from the dataset and manually corrected for backgrounds. Examples of raw data, correction spectra, and corrected datasets are shown in Fig. S1–S3.† Each image was then combined into one big dataset and analysed together with the N-FINDR spectral unmixing algorithm to locate cellular compartments.^24^ Three components representing lipids, cytoplasm, and nucleus were selected and fitted using non-negativity least squares fitting to all the images, which allowed the quantification of the content of each cell. The average abundance value for each component was then used to indicate the amount of each component in each cell type. Representative combined N-FINDR images are shown in Fig. 2A–C of triangular, square, and circular cells, respectively. Colours correspond to the corresponding spectra in Fig. 2D with trace 1 representing lipids (similar to triolein) in red, cytoplasm in green, and nucleus spectra in blue. Typical triolein like molecular features at 1266, 1301, 1440, 1655 and 1743 cm^−1^ characterised the red spectrum. The extracted green cytoplasm spectrum showed the typical features of cells such as Phenylalanine (1003, 1032, 1209), CH_2/3_ deformation of proteins and lipids (1303, 1335, 1444 cm^−1^), nucleotides (1577 cm^−1^), Amide III (1250 cm^−1^) and Amide I (1654 cm^−1^). In addition, there were also bands indicating Collagen (854, 873, 938 1243 cm^−1^). The nucleus spectra were characterised by higher concentrations of DNA (785, 1335, 1575 cm^−1^) and nucleic acids (1097 cm^−1^) and shifts in the Amide I (1663 cm^−1^) and CH2/3 Deformation (1453 cm^−1^) compared to the cytoplasm spectra. The mean abundance value for each component was calculated using non-negativity least squares fitting for a total of 12 images. The resulting mean abundance values are shown in Fig. 2E–G. These data clearly showed a trend to higher lipid content (Fig. 2E) in the circular cells and suggested a trend to higher protein content in square and triangular cells. In addition, the mean abundance values of the cytoplasm spectra suggested higher collagen content in square and triangular cells compared to circular cells. It is noteworthy that the low signal-to-noise ratio of the spectra in the wavenumber regions that could be applied to locate collagen I as well as the significant overlap between collagen bands and other proteins bands did not allow mapping of single collagen bands. However, the blue areas of the Raman spectroscopy maps indicated a higher protein content in the vicinity of the square and triangular cells that correlated with the location of collagen, and indicated that the main difference in protein content could be due to collagen. Using longer integration times to improve the signal-to-noise ratio in the future might address this problem.

### Immunohistochemical analysis

**Fig. 3:**
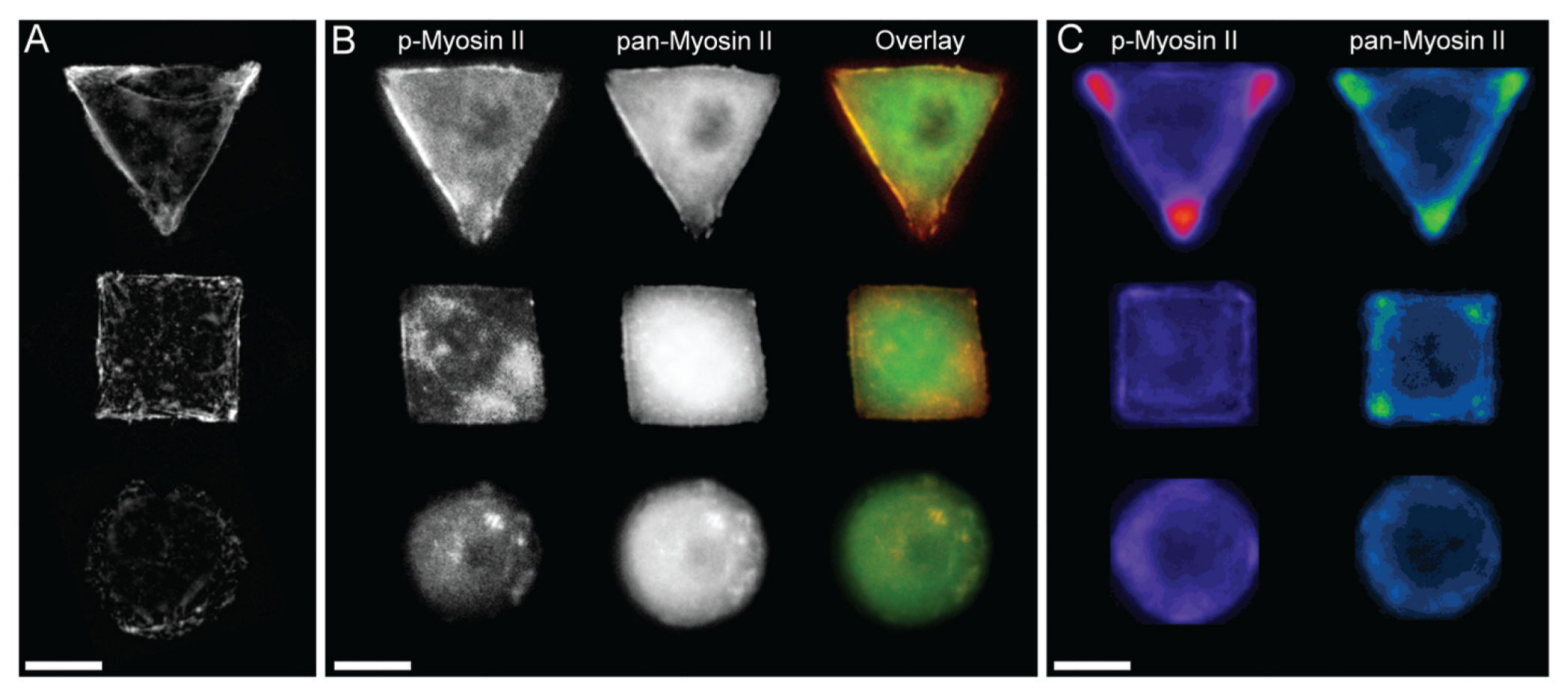
(A) Micropatterned hMSCs stained against F-actin after 24 hours incubation. Triangular and square shaped cells result in formation of large stress fibres on the cell perimeter spanning from on edge to another, while round cells show a cortical F-actin network with smaller fibres. (B) Micropatterned cells stained for myosin IIa show a similar trend in myosin fibre formation as observed by the cell shape dependent changes of actin cytoskeleton. The separate images as well as overlay of pan-myosin IIa (green) as well as phospho-myosin IIa (red) is shown. (C) Immunofluorescence intensity heat maps of >30 micropatterned single hMSCs stained for phosphorylated-myosin IIa and pan-myosin IIa. Higher intensity is represented by brighter colours. Scale bar = 20 µm.

**Fig. 4:**
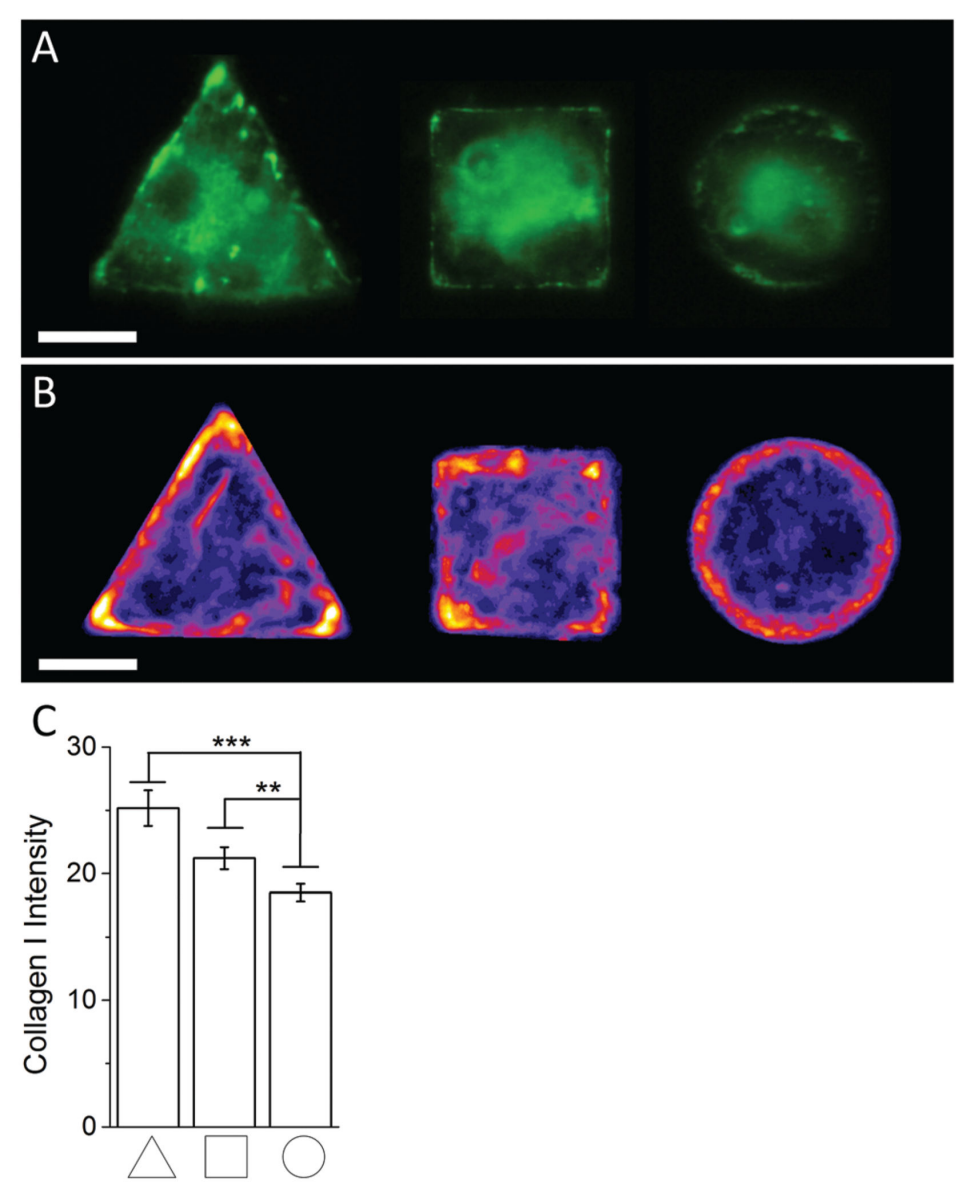
(A) Representative immunofluoresence images of micropatterned hMSCs stained against collagen I. (B) Immunofluoresence intensity heatmaps of triangular, square, and circular shaped micropatterned hMSCs stained against collagen I illustrate the previously observed localisation dependent signal intensity and overall collagen I abundance across the whole cell population quantitatively. Scale bar = 20 µm. (C) Immunofluorescence image quantification of the average signal intensity of micropatterned hMSCs stained against collagen I.

In order to investigate the findings obtained by Raman spectroscopy analysis, traditional immunohistochemical analysis was performed. hMSCs micropatterns stained against F-actin showed clear differences in cellular architecture between the different cell shapes. Square and triangular shaped cells resulted in a pronounced stress fibre formation from one edge to another, while circular shaped cells show a network of much smaller cortical actin fibres around the cellular perimeter (Fig. 3A). A similar trend in cell shape dependent cytoskeletal formation was observed when micropatterned hMSCs were co-immunolabelled against phosphorylated myosin IIa (red) and pan-myosin IIa (green) (Fig. 3B), which is an indicator of stress fibre formation and eventually stress fibre contraction.^25^,^26^ These results indicated increased actin and myosin fibre formation in triangular and square shaped cells compared to round cells, and correlated with a higher content of protein in the triangular and square cells. These observations were confirmed by generating fluorescence intensity heatmaps which provided information about intensity and localisation of phosphorylated and pan myosin IIa over the entire cell population (Fig. 3C). To generate immunofluorescent heatmaps, hMSCs cultured on the three different shapes (triangular, square, circular) were imaged at the same time using the same microscope and camera settings. Raw fluorescent images were background-subtracted using Image J software, incorporated into a Z Hyperstack, and the summarised intensity calculated for heatmap generation. Overall, these findings indicated that the higher protein content measured by Raman spectroscopy analysis could be an indicator of cytoskeletal changes in the cells. As cytoskeletal changes have been reported to have implications in stem cell differentiation and cancer development,^4^–^6^,^27^ our findings could represent an alternative analysis technique for regenerative medicine and drug screening. To further examine our Raman spectroscopy findings suggesting higher collagen content in triangular and square shaped cells compared to circular, total collagen I intensity of micropatterned hMSCs was quantified by immunofluorescence using standard procedures. Briefly, cells were fixed with 4% (v/v) formalin in dH_2_O (Sigma) for 15 minutes at room temperature, washed with PBS, permeabilised with 0.25% (v/v) Triton-X-100/PBS for 2 minutes, washed with PBS again, and then blocked with 4% (w/v) bovine serum albumin in PBS. Cells were incubated with Alexa Fluor® 488 phalloidin (1 : 300) for 30 minutes to stain for F-actin. Primary and secondary antibodies were incubated in blocking buffer for 1 hour at room temperature. Polyclonal rabbit anti Collagen I antibody (1 : 400) was purchased from Abcam. Alexa Fluor® 488, DAPI (4′,6-diamidino-2-phenylindole), and anti-rabbit Fluor® 488 (1 : 400) were obtained from Molecular Probes, Invitrogen. An Olympus BX51 system microscope inverted with a 40× objective was used to image fluorescence and phase contrast samples. Fluorescence microscopy images of cellular micropatterns stained against collagen type I showed the highest fluorescence intensity in triangular cells and lowest in circular cells. This result was consistent with the trend of cellular geometry inducing cell contractility. Furthermore, fluorescence images indicated a higher collagen I abundance around the perimeter of micropatterned cells (Fig. 4A). These observations were confirmed using fluorescence intensity heatmaps of collagen I (Fig. 4B) using the same procedure as described for Fig. 3C. The total collagen type I expression in micropatterns was determined quantitatively by immunofluorescence assay. The total collagen type I expression level per cell micropattern was quantified as follows. The immunofluorescence microscopy images of cells with different shapes were taken under the same conditions, including the same exposure time and gain factor. Cells that were fully spread on entire micro-islands, as confirmed by phase contrast images, were selected for analysis. The total average intensity per pixel of each cell micro-island was measured using ImageJ. To subtract background noise, the total average intensity of the area near the cellular boundary was used. Approximately 100 cells (*n* = 100) from 3 independent experiments were analysed for each shape. The measured fluorescence intensity in triangular and square shaped cells was significantly higher than circular cells (Fig. 4C). This observation was in line with our earlier observations that a higher collagen I expression in cellular geometry induced a higher cell contractility.

Together, this immunohistochemistry-based analysis of collagen I expression in micropatterned cells was in line with the results obtained by Raman spectroscopy mapping and suggested a higher collagen I content in triangular and square cells compared to circular. Given the increased cytoskeletal formation in these shapes, these findings point to an interesting connection between collagen I content and a cell’s cytoskeleton. This same observation has been made in previous studies.^28^–^32^ It is of note that our analysis only considered endogenous collagen I or adhered collagen I around the perimeter of the cells; it did not take into account collagen secreted into the cell medium. However, it has been previously reported that hMSCs derived from bone marrow showed a negligible amount of collagen I secretion into the cell medium.^33^ Our findings also showed that Raman spectroscopy analysis can provide quantitative information about specific molecules in micropatterned cells without the need to label the molecules beforehand. Since Raman spectroscopy analysis can potentially be performed on live cells using different excitation wavelengths,^34^ this technique holds great promise for a variety of applications. For example, this technology could be used in regenerative medicine to track stem cell lineage commitment *in vitro*. This analysis could also be used to assess the effect of chemicals or new tissue engineering scaffolds over time.

## Conclusions

We report a new approach using high resolution Raman spectroscopy mapping of a micro-engineered stem cell platform. Using this technology, we obtained quantitative information about the concentration of individual intracellular molecules such as proteins, lipids, and a variety other metabolites within cells depending on their architecture and cytoskeletal arrangement. We believe this analysis technique is highly relevant for *in vitro* drug screening applications and regenerative medicine. We would like to gratefully acknowledge the Wellcome Trust Senior Investigator Grant “Exploring and Engineering the Cell-Material Interface for Regenerative Medicine” (098411/Z/12/Z) along with the UK Regenerative Medicine Platform Hub “Engineering and Exploiting the Stem Cell Niche” (MR/K026666/1), which is funded by the Medical Research Council, the Engineering and Physical Sciences Research Council and the Biotechnology and Biological Sciences Research Council, for their generous support. Martin A. B. Hedegaard was partially supported by The Danish Council for Independent Research (FTP contract no. 0602-02350B).

## Supplementary Material

† Electronic supplementary information (ESI) available. See DOI: 10.1039/c4an02346c

ESI
