# Supplementary material for: High resolution Raman spectroscopy mapping of stem cell micropatterns†
Source: Analyst. Author manuscript; Available in PMC 2017 Apr 27. (PMC5407440; doi:10.1039/c4an02346c)
Supplement: ESI [file NIHMS72466-supplement-ESI.pdf]

Electronic Supplementary Material (ESI) for Analyst.
This journal is © The Royal Society of Chemistry 2015

Supplementary Material:

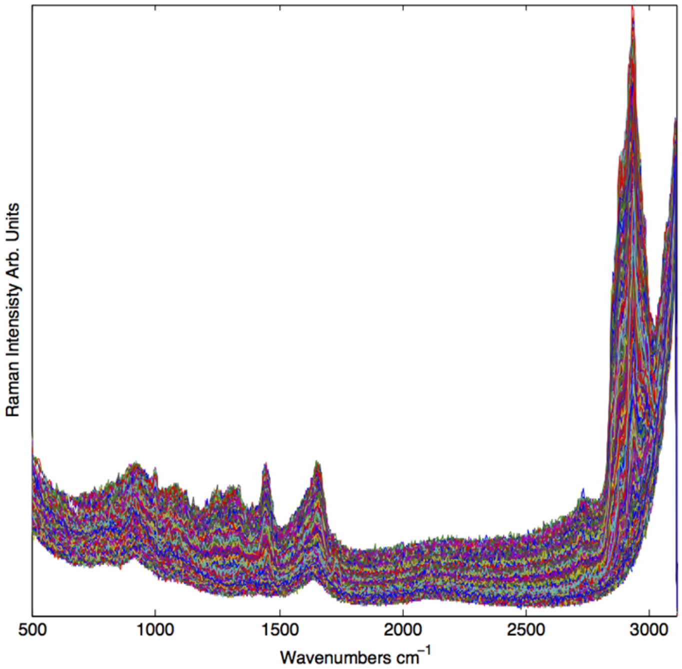


Figure S1: Example of Raw data before Extended Multiplicative Scater Correction and Spectral Interference Subtraction (EMSC-SIS), Correction spectra.

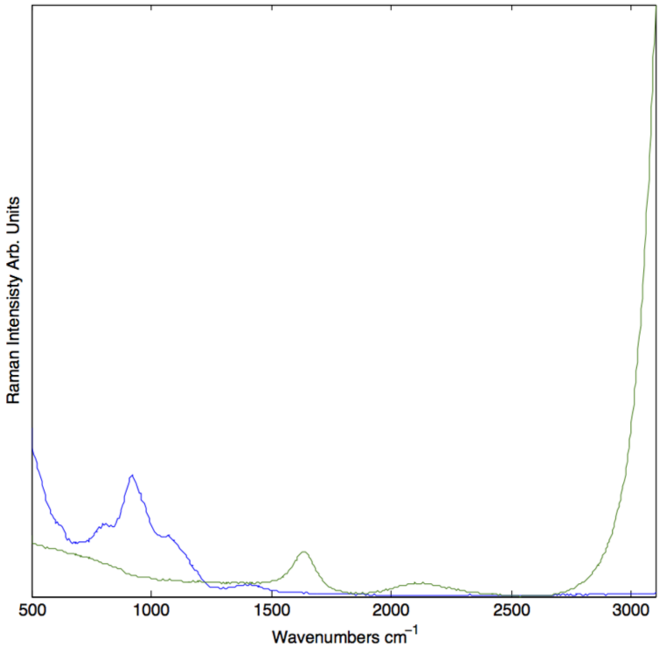


Figure S2: Spectra of water (green) and glass (blue) used for correction of backgrounds.

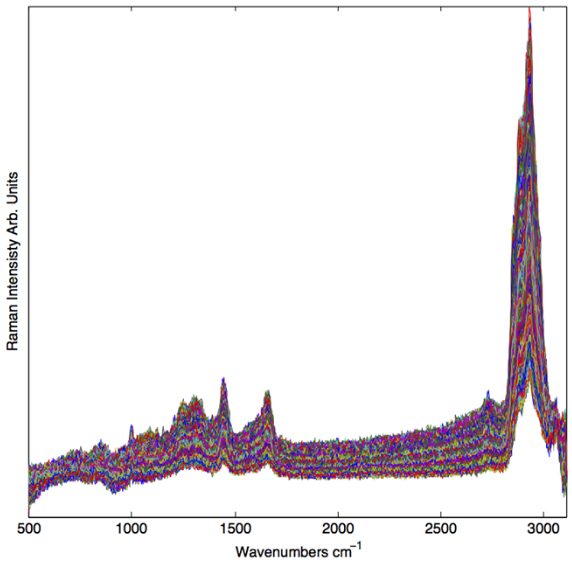


Figure S3: Dataset after correction using EMSC-SIS.
